# Randomized, crossover, double-blind, placebo-controlled trial to assess the lipid lowering effect of co-formulated TDF/FTC

**DOI:** 10.7448/IAS.17.4.19550

**Published:** 2014-11-02

**Authors:** José Ramón Santos, María Saumoy, Adrian Curran, Isabel Bravo, Jordi Navarro, Carla Estany, Daniel Podzamczer, Esteban Ribera, Eugenia Negredo, Bonaventura Clotet, Roger Paredes

**Affiliations:** 1 Internal Medicine Department, Lluita contra la SIDA Foundation, Hospital Universitari Germans Trias i Pujol, Barcelona, Spain; 2 HIV Unit, Infectious Disease Service, Bellvitge University Hospital, Bellvitge Biomedical Research Institute, Hospitalet de Llobregat, Spain; 3 Infectious Diseases Department, Hospital Universitari Vall d'Hebron, Universitat Autònoma de Barcelona, Barcelona, Spain; 4 Lluita contra la SIDA Foundation, Hospital Universitari Germas Trias i Pujol, Internal Medicine Department, Universitat de Vic, Barcelona, Spain; 5 Internal Medicine Department, Hospital Universitari Germans Trias i Pujol, IrsiCaixa Foundation, Universitat de Vic, Barcelona, Spain

## Abstract

**Introduction:**

Previous studies have described improvements on lipid parameters when switching from other antiretroviral drugs to tenofovir (TDF) and impairments in lipid profile when discontinuing TDF. [1–3] It is unknown, however, if TDF has an intrinsic lipid-lowering effect or such findings are due to the addition or removal of other offending agents or other reasons.

**Materials and Methods:**

This was a randomized, crossover, double-blind, placebo-controlled clinical trial (NCT 01458977). Subjects with HIV-1 RNA <50 copies/mL during at least 6 months on stable DRV/r (800/100 mg QD) or LPV/r (400/100 mg BID) monotherapy, with confirmed fasting total cholesterol ≥200 or LDL-cholesterol ≥130 mg/dL and not taking lipid-lowering drugs were randomized to (A) adding TDF/FTCduring 12 weeks followed by 24 weeks without TDF/FTC, or (B) continuing without TDF/FTC for 12 weeks, adding TDF/FTC for 12 weeks and then withdrawing TDF/FTC for 12 additional weeks. Randomization was stratified by DRV/r or LPV/r use at study entry. All subjects received a specific dietary counselling. Primary endpoints were changes in median fasting total, LDL and HDL-cholesterol 12 weeks after TDF/FTC addition. Analyses were performed by ITT.

**Results:**

46 subjects with a median age of 43 (40–48) years were enrolled in the study: 70% were male, 56% received DRV/r and 44% LPV/r. One subject withdrew the study voluntarily at week 4 and another one interrupted due to diarrhoea at week 24. Treatment with TDF/FTC decreased total, LDL and HDL-cholesterol from 235.9 to 204.9 (p<0.001), 154.7 to 127.6 (p<0.001) and 50.3 to 44.5 mg/dL (p<0.001), respectively. In comparison, total, LDL and HDL-cholesterol levels remained stable during placebo exposure. Week 12 total cholesterol (p<0.001), LDL-cholesterol (p<0.001) and HDL-cholesterol (p=0.011) levels were significantly lower in TDF/FTC versus placebo. Treatment with TDF/FTC reduced the fraction of subjects with abnormal fasting total-cholesterol (≥200 mg/dL) from 86.7% to 56.8% (p=0.001) and LDL-cholesterol (≥130 mg/dL) from 87.8% to 43.9% (p<0.001), which was not observed with placebo. There were no virological failures, and CD4 and triglyceride levels remained stable regardless of exposure.

**Conclusion:**

Coformulated TDF/FTC has an intrinsic lipid-lowering effect, likely attributable to TDF.
